# Brain biopsy and metagenomic sequencing enhance aetiological diagnosis of encephalitis

**DOI:** 10.1093/braincomms/fcaf165

**Published:** 2025-04-28

**Authors:** Yusuke Sakiyama, Jun-Hui Yuan, Akiko Yoshimura, Mika Takeuchi, Yoshimitsu Maki, Takuma Mori, Jun Takei, Masahiro Ando, Yu Hiramatsu, Satoshi Nozuma, Yujiro Higuchi, Hajime Yonezawa, Mari Kirishima, Masayuki Suzuki, Takahiro Kano, Monami Tarisawa, Shunta Hashiguchi, Misako Kunii, Shoki Sato, Ikuko Takahashi-Iwata, Akihiro Hashiguchi, Eiji Matsuura, Shuji Izumo, Akihide Tanimoto, Hiroshi Takashima

**Affiliations:** Department of Neurology and Geriatrics, Kagoshima University Graduate School of Medical and Dental Sciences, Kagoshima, 890-8520, Japan; Department of Neurology and Geriatrics, Kagoshima University Graduate School of Medical and Dental Sciences, Kagoshima, 890-8520, Japan; Department of Neurology and Geriatrics, Kagoshima University Graduate School of Medical and Dental Sciences, Kagoshima, 890-8520, Japan; Department of Neurology and Geriatrics, Kagoshima University Graduate School of Medical and Dental Sciences, Kagoshima, 890-8520, Japan; Department of Neurology, Kagoshima City Hospital, Kagoshima, 890-8760, Japan; Department of Neurology and Geriatrics, Kagoshima University Graduate School of Medical and Dental Sciences, Kagoshima, 890-8520, Japan; Department of Neurology and Geriatrics, Kagoshima University Graduate School of Medical and Dental Sciences, Kagoshima, 890-8520, Japan; Department of Neurology and Geriatrics, Kagoshima University Graduate School of Medical and Dental Sciences, Kagoshima, 890-8520, Japan; Department of Neurology and Geriatrics, Kagoshima University Graduate School of Medical and Dental Sciences, Kagoshima, 890-8520, Japan; Department of Neurology and Geriatrics, Kagoshima University Graduate School of Medical and Dental Sciences, Kagoshima, 890-8520, Japan; Department of Neurology and Geriatrics, Kagoshima University Graduate School of Medical and Dental Sciences, Kagoshima, 890-8520, Japan; Department of Neurosurgery, Kagoshima University Graduate School of Medical and Dental Sciences, Kagoshima, 890-8520, Japan; Department of Pathology, Kagoshima University Graduate School of Medical and Dental Sciences, Kagoshima, 890-8520, Japan; Division of Neurology, Department of Medicine, Jichi Medical University, Tochigi, 329-0498, Japan; Department of Neurology, Obihiro Kosei General Hospital, Obihiro, 080-0024, Japan; Department of Neurology, Obihiro Kosei General Hospital, Obihiro, 080-0024, Japan; Department of Neurology and Stroke Medicine, Yokohama City University Graduate School of Medicine, Yokohama, 236-0004, Japan; Department of Neurology and Stroke Medicine, Yokohama City University Graduate School of Medicine, Yokohama, 236-0004, Japan; Department of Neurology, Faculty of Medicine and Graduate School of Medicine, Hokkaido University, Sapporo, 060-8648, Japan; Department of Neurology, Faculty of Medicine and Graduate School of Medicine, Hokkaido University, Sapporo, 060-8648, Japan; Department of Neurology and Geriatrics, Kagoshima University Graduate School of Medical and Dental Sciences, Kagoshima, 890-8520, Japan; Department of Neurology and Geriatrics, Kagoshima University Graduate School of Medical and Dental Sciences, Kagoshima, 890-8520, Japan; Department of Neurology and Geriatrics, Kagoshima University Graduate School of Medical and Dental Sciences, Kagoshima, 890-8520, Japan; Department of Pathology, Kagoshima University Graduate School of Medical and Dental Sciences, Kagoshima, 890-8520, Japan; Department of Neurology and Geriatrics, Kagoshima University Graduate School of Medical and Dental Sciences, Kagoshima, 890-8520, Japan

**Keywords:** encephalitis, metagenomics, next-generation sequencing, brain, archaea

## Abstract

Identifying the aetiology of CNS diseases, regardless of their infectious or non-infectious nature, is often intricate. Next-generation sequencing (NGS) has emerged as a powerful tool for sensitive and unbiased screening of tissue or body fluid specimens. This study aimed to investigate the underlying aetiology of patients with suspected infectious CNS diseases. Between April 2013 and October 2021, we collected brain tissue samples from 33 patients diagnosed with encephalitis or encephalitis-like CNS diseases, obtained via biopsy or autopsy, and underwent metagenomic NGS (mNGS) in conjunction with pathological evaluations. Moreover, we employed PCR-based assays and pathogen-specific immunostaining to corroborate the presence of pathogens within the tissue samples. Among the 33 patients, mNGS elucidated pathogen-specific genomic sequences in 7 cases (21.2%), including halobacteria (archaea), *Balamuthia mandrillaris*, Epstein–Barr virus, *Toxoplasma gondii* and herpes simplex virus. Additionally, brain tissue mNGS ruled out known pathogens, identifying 14 cases (42.4%) of non-infectious CNS diseases, which included neoplastic, autoimmune/inflammatory and amyloid angiopathy conditions. The adjustment of therapeutic strategies based on these findings led to improvements in clinical symptoms, imaging outcomes and patient prognosis. Brain biopsy serves as both a direct pathological research target and a valuable source of samples for unbiased high-throughput sequencing. Our study illustrates the reliability of mNGS on brain tissue, which significantly improves the diagnostic rate for suspected encephalitis or encephalitis-like diseases of unknown aetiology. These findings underscore the importance of mNGS in guiding more precise and effective therapeutic interventions for patients in clinical practice.

## Teaser

Sakiyama *et al*. reported the application of metagenomic next-generation sequencing on brain tissue samples (mNGS-brain) in 33 patients suspected of encephalitis, successfully identifying aetiologies in 21.2% of cases and excluding infectious causes in an additional 42.4%. These findings provide compelling evidence for the promising role of mNGS-brain in the aetiological diagnosis of encephalitis.

## Graphical Abstract

**Graphical Abstract:**
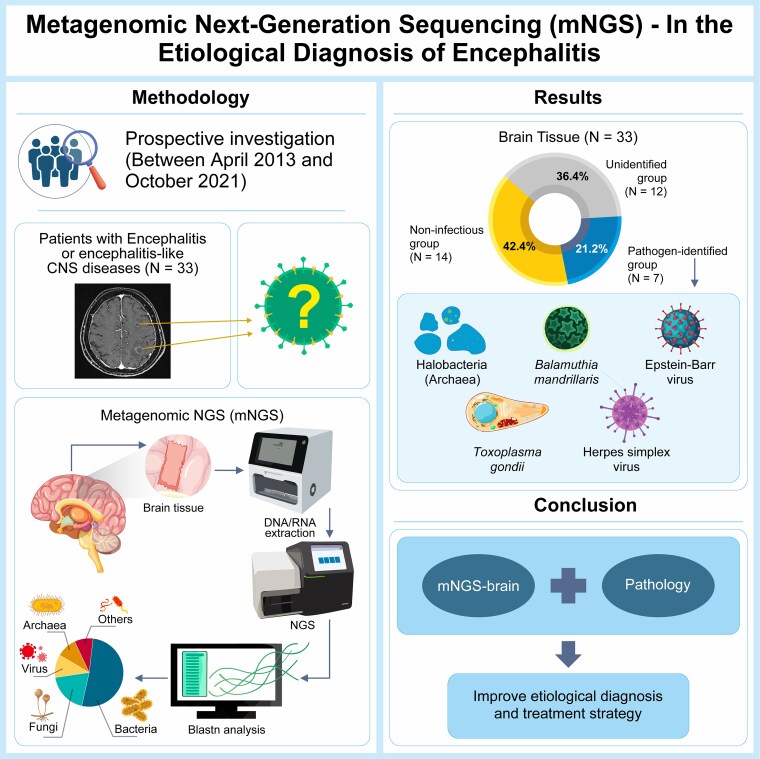

## Introduction

The accurate and prompt diagnosis of infectious CNS diseases represents a substantial challenge in clinical medicine. These conditions, encompassing encephalitis and meningitis, are frequently characterized by inflammation of the brain or its surrounding membranes, resulting from a diverse spectrum of infectious agents, such as bacteria, viruses, fungi and parasites. Conventional diagnostic approaches, such as culture-based techniques and PCR-based assays, have been employed for many years but are often hindered by their limited applicability and sensitivity, particularly when dealing with infrequent or novel pathogens. Consequently, ∼50% of encephalitis cases remain undiagnosed.^1,2^

In cases of encephalitis or encephalitis-like CNS diseases where patients experience unexplained clinical deterioration or fail to respond to empirical treatment, brain biopsy is occasionally performed as a last resort. Despite its invasive nature, pathological analysis of biopsy samples often provides limited diagnostic clarity, with non-specific inflammatory changes reported in ∼39% of cases.^3^ These cases are commonly categorized as ‘encephalitis not otherwise specified’ (ENOS), further underscoring the diagnostic limitations of traditional methods.^4^

Recently, metagenomic next-generation sequencing (mNGS) has emerged as a promising tool with the potential to revolutionize the diagnosis of infectious diseases. It enables the simultaneous identification of a diverse array of pathogens through a single run.^5-7^ For CNS infections, CSF analysed with mNGS provides a non-invasive and reproducible method for detecting pathogen DNA and RNA, streamlining the diagnostic process. Its clinical utility has been demonstrated in large-scale studies. However, in a prospective study of 204 cases, mNGS failed to identify the pathogen in 26 of 58 confirmed infections, including 7 detected in non-CSF samples and 8 with low pathogen levels in CSF.^8^

In contrast, mNGS applied to brain tissue samples (hereafter referred to as mNGS-brain) has been reported primarily in case studies and reviews,^9,10^ with large-scale investigations remaining rare.^11^ In this study, we aimed to evaluate the diagnostic utility of mNGS-brain in 33 patients clinically diagnosed with encephalitis or encephalitis-like CNS diseases. Our findings highlight the potential of mNGS-brain to improve diagnostic certainty, successfully identifying aetiologies in 21.2% of cases and contributing to the exclusion of infectious causes in an additional 42.4% of patients.

## Materials and methods

### Study design and sample collection

**Figure 1:**
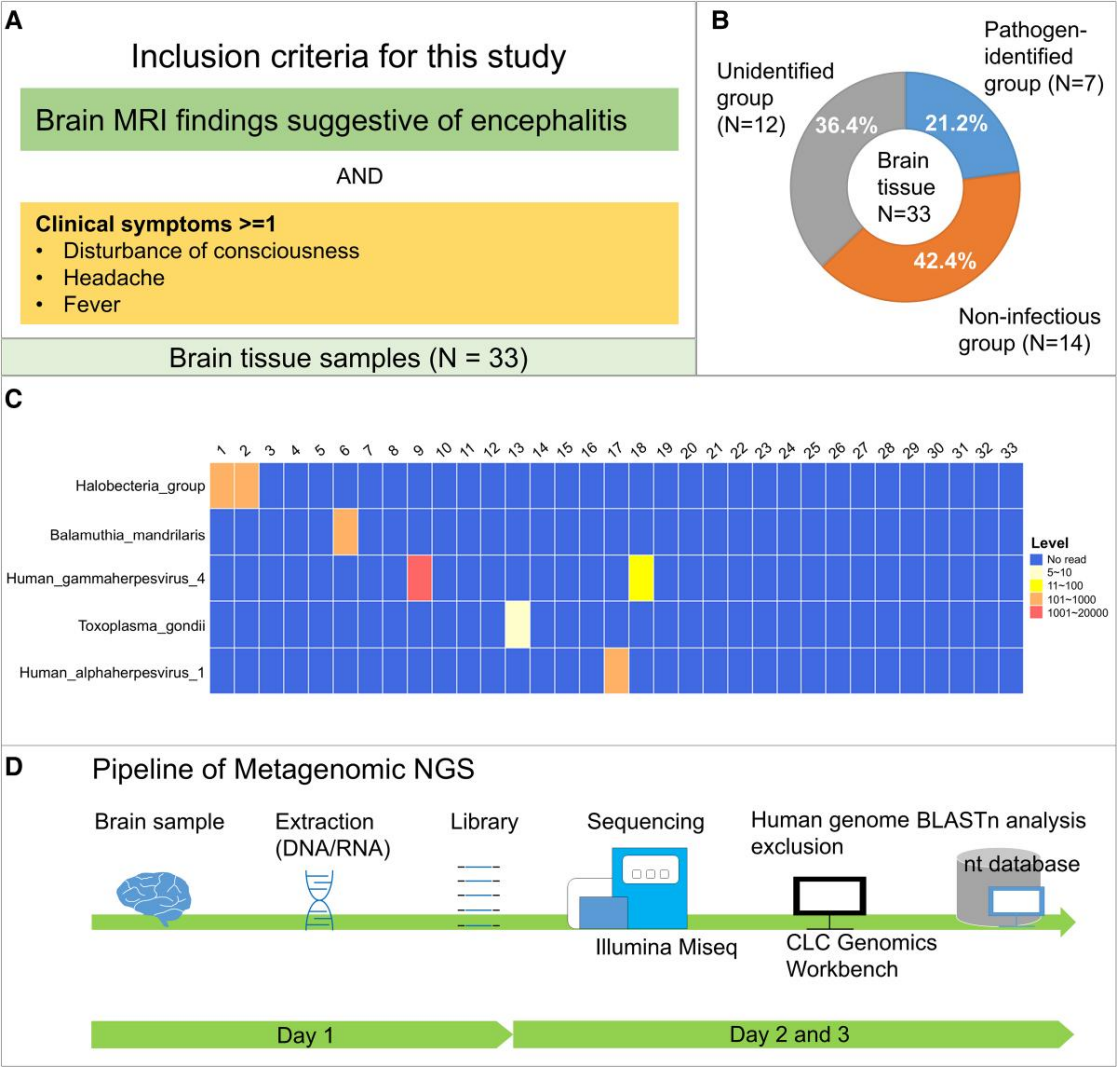
**Research overview and mNGS findings.** (**A**) Inclusion criteria for patients (*n* = 33) with encephalitis and encephalitis-like CNS diseases in this study. (**B**) mNGS identifies aetiologies in ∼21.2% of cases and contributing to the exclusion of infectious causes in an additional 42.4% of patients. The aetiology remains unidentified in 36.4% of cases. (**C**) Heatmap showing the pathogens detected using mNGS. *X*-axis: the sample numbers. (**D**) Pipeline of mNGS.

This study was designed as a prospective investigation, enrolling patients who received clinical diagnoses of encephalitis or encephalitis-like CNS diseases. The inclusion criteria were pre-defined as follows: (i) presence of indicative abnormalities observed on brain MRI consistent with encephalitis and (ii) ≥1 of the following conditions: disturbance of consciousness, headache and fever (Fig. 1A).

Between April 2013 and October 2021, we collected brain tissue samples from a total of 33 patients across 9 participating facilities (Supplementary Fig. 1). Among these brain samples, 30 and 3 samples were obtained through biopsy and autopsy procedures, respectively.

The Ethics Committee of Kagoshima University Hospital approved this study, which aimed to investigate the aetiology of encephalitis and meningitis in patients (Approval No. 492).

### DNA/cDNA isolation from brain tissues and shotgun metagenomic next-generation sequencing-brain analysis

The experimental procedures are described in Fig. 1D.

#### Brain tissue collection and DNA/cDNA isolation

The brain biopsy/autopsy samples were procured by experienced neurosurgeons or pathologists, either at local medical facilities or within our hospital. These brain tissues were carefully separated and promptly placed into sterile containers that had been pre-treated with sterile saline solution. Subsequently, the samples were transported to our laboratory. Upon arrival, the samples underwent immediate processing for DNA and RNA isolation procedures. In cases where immediate processing was not feasible, the samples were stored at −80°C.

From April 2013 to April 2020, DNA was extracted from brain tissue samples using the DNeasy Blood and Tissue Kit (Qiagen). After May 2020, both DNA and RNA were concurrently extracted from brain tissue samples using MagMAX^TM^ Total Nucleic Acid Isolation Kit and Dynabeads (Thermo Fisher Scientific, Waltham, MA, USA) through a KingFisher Duo Prime Purification System (Thermo Fisher Scientific), following the manufacturer’s instructions.

The RNA was subjected to reverse transcription using SuperScript IV Reverse Transcriptase (Thermo Fisher Scientific) with non-ribosomal random hexamers, according to a previously described protocol.^12^ For the second strand synthesis, a reaction mixture containing 20 µl of single-strand cDNA, 1 µl of Klenow fragment (3′-5′ exo-) and 1 µl of RNase H was incubated at 37°C for 30 min, followed by an additional incubation at 75°C for 20 min.

#### Shotgun metagenomic next-generation sequencing-brain

Library preparation was conducted using the Illumina DNA Prep Kit and protocol, following previously described methods.^13^ Briefly, DNA or cDNA samples were fragmented by transposon-based approach and then ligated with adapters. Size selection was performed using magnetic beads to eliminate small fragments, followed by 5–12 cycles of PCR amplification using adapter-specific primers. Equimolar concentrations of an average of four DNA or cDNA libraries with different adaptors were pooled together (two to three cases were analysed simultaneously). The resulting libraries were combined in equimolar concentrations. For sequencing, a total of 50 pmol of the pooled DNA/cDNA library was subjected to sequencing on the Illumina MiSeq system, utilizing the Illumina Miseq v2 Reagent Kit. Sequencing was conducted in a paired-end mode (150 bp ×2), producing ∼15 million reads per run. To compensate the limited base diversity in the libraries, 10% PhiX was included as a control during each sequencing run. Autoclaved water was applied as a negative control sample.

#### Next-generation sequencing data processing for pathogen identification

To eliminate homologous reads derived from the human genome, the high-quality sequencing data underwent alignment against the human genome database (hg38; University of California, Santa Cruz (UCSC)) and the human mRNA database (RefSeq release 54) using the ‘CLC Genomics Workbench’ software (QIAGEN, Hilden, Germany). For the unmapped reads that remained after alignment, we conducted a search for similar species within the National Center for Biotechnology Information nucleotide collection database via Nucleotide BLAST (BLASTn). A comprehensive quality control assessment was employed, including an E-value cut-off of 1e−20, a minimum match rate of 80%, an alignment rate of 80% or higher and a minimum hit length of 80 bases. The determination of species-specific similarity for each read was achieved through the utilization of a proprietary pipeline developed in collaboration with the Maze Inc. This analysis pipeline typically operated within a time frame of <12 h for each sample.

To ensure the elimination of reproducible false-positive artefacts, we implemented the following exclusion criteria for BLASTn-based similarity matches: (i) reads that collectively mapped to specific regions of a pathogen genome sequence; (ii) matched fragments that were also detected in autoclaved water sample; and (iii) similarity matches that were detected in more than 20% of all samples. These criteria were put in place to enhance the specificity and reliability of our analysis by filtering out potentially erroneous or non-specific matches. Furthermore, read counts of <5 for all species were excluded from pathogen candidates.

### Pathological analysis of brain tissue samples

Tissue sections underwent a standard haematoxylin–eosin (HE) staining procedure, enabling the assessment of overall tissue morphology and cellular structure. Periodic acid-Schiff (PAS) staining was conducted to identify the presence of glycogen and mucin substances within the tissue. Immunohistochemical staining was carried out to evaluate the expression of specific proteins, and the following antibodies were utilized: CD, MUM1, Bcl-6, Olig2, MIB-1, PLAP and c-kit. Epstein–Barr encoding region (EBER) *in situ* hybridization was performed to detect the Epstein–Barr virus (EBV) in tissue sections.

### Literature review strategy

A systematic search of the PubMed database was conducted to investigate studies utilizing mNGS on brain samples from patients with encephalitis. The detailed search strategy, covering publications up to September 2024, is provided in the Supplementary material. This search identified 24 papers, encompassing 32 relevant cases (Table 1; Supplementary Table 1).^10,14-36^ Among these cases, 16 involved post-mortem examinations, 9 resulted in death, 3 lacked reported outcomes and 4 showed clinical improvement.

**Table 1:** Literature review of shotgun mNGS for brain samples from patients with encephalitis

Citation	Pathogen	Reads of pathogen	Confirmatory test	Diagnosis
Quan *et al*.^14^	Astrovirus	12	PCR and IH	Astrovirus encephalitis
Chan *et al*.^15^	Measles virus	1067	PCR	SSPE
	HSV1	10	PCR	HSV1 encephalitis
Brown *et al*.^16^	Astrovirus	46	PCR, IH and EM	Astrovirus encephalitis
Frémond *et al*.^17^	Astrovirus	15 contigs	PCR	Astrovirus encephalitis
Greninger *et al*.^18^	*B. mandrillaris*	30	PCR and pathology	Primary amoebic meningoencephalitis
Naccache *et al*.^19^	Astrovirus	1612	PCR and IH	Astrovirus encephalitis
Lum *et al*.^20^	Astrovirus	Not reported	PCR	Astrovirus encephalitis
Morfopoulou *et al*.^21^	HCoV-OC43	1 000 000	PCR and IH	HCoV-OC43 encephalitis
Salzberg *et al*.^10^	JC polyomavirus	8944	Pathology and IH	PML
	*M. tuberculosis*	15	Pathology	Granuloma, tuberculosis
	EBV	18	ISH	EBV encephalitis
Morfopoulou *et al.*^22^	Mumps vaccine strain	77 624	PCR and IH	Chronic encephalitis caused by mumps vaccine stain
Wilson *et al*.^23^	Cache Valley virus	2	PCR and IH	Chronic viral meningoencephalitis
Lipowski *et al*.^24^	Tick-borne encephalitis virus	988	PCR	Tick-borne encephalitis
Osterman *et al*.^25^	HSV1	490	PCR	HSV1 encephalitis
Normandin *et al*.^26^	Powassan virus	Not reported	PCR and IH	Fatal encephalitis
Rodriguez *et al*.^27^	Measles virus	>4 800 000	PCR	Measles inclusion-body encephalitis
Tuddenham *et al*.^28^	Human pegivirus	4	PCR	Enchephalitis (aetiology unknown)
Nilsson *et al*.^29^	HCoV-OC43	300 000	PCR	HCoV-OC43 encephalitis
Pérot *et al*.^30^	Umbre orthobunyavirus	2029	PCR and ISH	Lethal encephalitis
Howard-Jones *et al*.^31^	Japanese encephalitis virus	Not reported	PCR and IgM	Japanese encephalitis
Regnault *et al*.^32^	European bat lyssavirus Type 1	90% coverage of reference genome	PCR and EM	Lethal encephalitis
Maamary *et al*.^33^	Japanese encephalitis virus	Not reported	IgM	Japanese encephalitis
Gould *et al*.^34^	Yellow fever vaccine virus	15	ISH	Encephalitis caused by yellow fever vaccine stain
Piantadosi *et al*.^35^	JC polyomavirus	19	PCR	PML suspected
Guo *et al*.^36^	*Schistosoma japonicum*	Not reported	Pathology	Cerebral schistosomiasis

HCoV, human coronavirus; SSPE, subacute sclerosing panencephalitis; PML, progressive multifocal leukoencephalopathy; IH, immunohistochemistry; ISH, *in situ* hybridization.

## Results

### Clinical summary

Among the 33 patients included in this study (15 males and 18 females), the average age was 59.8 years, with an age range spanning from 14 to 89 years. The most prevalent symptom was a disturbance in consciousness, which was observed in 54.5% of all patients (18 cases), followed by fever in 21.2% (7 cases), headache in 18.2% (6 cases) and meningeal irritation sign in 6% (2 cases) of patients. CSF records were available for all 33 cases, and elevated white cell counts (≥5 cells/µl) were identified in 19 cases. Abnormal protein (≥50 mg/dl) and glucose levels (<50 mg/dl) were noted in 26 and 5 cases, respectively. The disease duration of these patients, from onset to the time of mNGS analysis, exhibited a wide range, spanning from 24 to 3615 days.

Prior to the mNGS-brain analysis, the 33 patients were further clinically classified as encephalitis with meningitis (12 cases), encephalitis without meningitis (14 cases), malignancy (4 cases) and demyelinating diseases (3 cases).

### Verification of method using positive controls

To verify the functionality of our workflow and data analysis pipeline, we conducted preliminary verification using multiple low-pathogenicity vaccine strains as positive controls. The selected vaccine strains included varicella-zoster virus, rubella virus and mumps orthorubulavirus. Following data alignment, the sequencing achieved nearly 100% coverage of the reference genomes for these viruses (Supplementary Fig. 2).

### Metagenomic next-generation sequencing-brain summary and case categorization

Using mNGS on DNA (33 cases) and cDNA (15 cases) extracted from brain tissue samples, we obtained a median read count of 6.3 M (range: 0.3–18.8 M) for DNA libraries and 6.2 M (range: 4.6–10.0 M) for cDNA libraries per sample. Following the removal of low-quality reads and human genome–derived sequences, the BLAST-based similarity analysis successfully identified pathogen-specific genomic sequences in seven cases afflicted with infectious CNS diseases (Fig. 1B).

Cases were classified into three groups based on a cross-referencing strategy that integrated all clinical and mNGS findings. (i) Pathogen-identified group: infectious pathogens were detected by mNGS analysis, with the diagnosis supported by the clinical course, drug susceptibility testing and pathological findings. (ii) Non-infectious group: no infectious pathogens were detected by mNGS analysis, and subsequent experimental findings suggested a non-infectious aetiology. (iii) Unidentified group: no specific cause identified despite all available analyses. The categorization process was carried out by two board-certified neurologists, with final decisions made in consultation with the attending physicians. The diagnosis of autoimmune encephalitis was based on the guidelines proposed in 2016.^37^

### Encephalitis with infectious pathogens

Within seven brain samples, the presence of various micro-organisms was detected: halobacteria (PT-1 and 2),^13^  *Balamuthia mandrillaris* (PT-6),^38^ EBV (PT-9 and 18), *Toxoplasma gondii* (PT-13) and herpes simplex virus (HSV) (PT-17). All clinical, CSF and mNGS data of these patients are summarized in Table 2.

**Table 2:** Clinical information of seven cases with pathogens detected by brain tissue metagenomic NGS

Case	Age	Main symptoms	Initial diagnosis	CSF-protein (mg/dl)	CSF-cell (/µl)	CSF-glucose (mg/µl)	Duration onset-mNGS	Brain frozen sample	mNGS-total reads	mNGS-suspected pathogen	Confirmatory testing	Diagnosis	Treatment	Clinical course
PT-1	50s	Dementia, parkinsonism and psychosis	Panic disorder	74	63	56	8 months	Biopsied (LMD)	7 335 306	Halobacteria: 126 reads	EM	Archaeal encephalitis susp.	SMX, trimethoprim and prednisolone	Improvement
PT-2	70s	Dementia, frontal lobe dysfunction and cerebellar ataxia	Unknown dementia	45	7	59	12 months	Biopsied (LMD)	303 736	Halobacteria: 130 reads	EM	Archaeal encephalitis susp.	SMX, trimethoprim and prednisolone	Died
PT-6	60s	Visual field loss, low-grade fever and general malaise	Sarcoidosis	77.8	43	86	3 months	Biopsied	9 390 554	*B. mandrillaris*: 129 reads	PCR IHS	Granulomatous amoebic encephalitis	Azithromycin, SMX and trimethoprim	Died
PT-9	50s	Headache, vomit, cerebellar ataxia and AIDS	Toxoplasma encephalitis or malignant lymphoma	48.9	3	49	1 month	Biopsied	12 599 876	EBV: 9806 reads	EBER-IHS	EBV-positive DLBCL	Radiation	Partial improvement
PT-13	80s	General fatigue and nausea	Unknown encephalitis	179.8	8	43	4 months	Post-mortem	3 615 402	*T. gondii*: 8 reads CMV: 2 reads	Nested-PCR histological staining	Toxoplasma encephalitis and CMV pneumonia		Post-mortem sample
PT-17	70s	Post-treatment of HSE, gait disturbance and dysarthria	Recurrence of HSE or malignant lymphoma	94	11	70	2 months	Biopsied	5 238 910	Human alphaherpesvirus 1: 175 reads	None	Recurrence of HSE and NMDAR encephalitis	Acyclovir, prednisolone and IVIg	Partial improvement
PT-18	60s	Decline in thinking ability and loss of appetite	Malignant lymphoma	156	188	58	1 month	Biopsied	7 924 566	EBV: 11 reads	None	EBV encephalitis susp.	Ganciclovir	Improvement

PT, patient; LMD, laser microdissection; EBV, Epstein–Barr virus; EM, electron microscopy; IHS, immunohistochemical staining; IVIg, intravenous immunoglobulin.

### Halobacterial encephalitis (PT-1 and 2)

**Figure 2:**
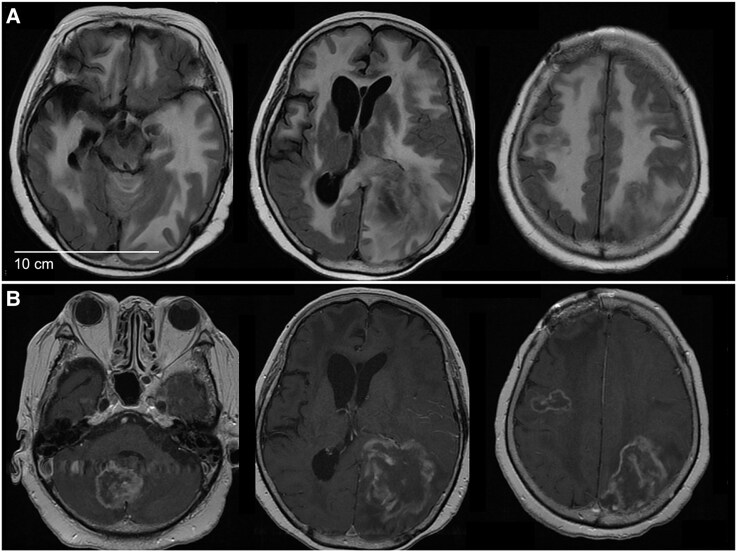
**Brain MRI of PT-2 with archaeal infection in advanced stage.** (**A**) DWI shows tumour-like lesions in the white matter of the right frontal and left parietal lobes with high signal intensity in the periphery and low signal intensity in the centre; FLAIR images reveal diffuse hyper-intensity in the white matter and a vague low-signal lesion with midline shift in the left parietal lobe. (**B**) Ce-T1WIs show enhancement at the margins of the lesion, consistent with the lesion seen on DWI. DWI, diffusion-weighted imaging; FLAIR, fluid attenuated inversion recovery; Ce-T1WIs, contrast-enhanced T1-weighted images.

Between 2005 and 2012, we studied four individuals residing within a 30-km radius in South Kyushu, Japan. These cases have been previously reported.^13^ Briefly, all patients presented with progressive dementia, tongue dyskinesia and abnormal signals within the medial temporal lobe in brain MRI. Neuropathological analysis revealed perivascular PAS-positive macrophages and round/oval bodies (diameter: 2–7 mm), without nuclei or cell walls. Through mNGS-brain analysis on two of these individuals (PT-1 and 2), we detected non-human sequences that matched with the halobacteria group (also known as halophilic archaea). All cases responded positively to trimethoprim-sulphamethoxazole (TMP-SMX) combined with corticosteroids. PT-2 was unable to continue TMP-SMX therapy due to hepatic impairment and, unfortunately, passed away 4 years after the diagnosis due to brain damage. An advanced stage brain MRI from Case 2 is presented in Fig. 2.

### Granulomatous amoebic encephalitis (PT-6)

A 60-year-old Japanese female presented with necrotizing encephalitis, and a brain biopsy revealed epithelioid granulomas with an unknown origin. Upon mNGS-brain analysis, a total of 129 genomic fragments belonging to *B. mandrillaris* were detected. Subsequently, immunopositive structures indicative of *B. mandrillaris* were observed in brain tissue, and the presence of amoebic trophozoites was confirmed through a skin biopsy.^38^

### Epstein–Barr virus–positive diffuse large B-cell malignant lymphoma (PT-9)

**Figure 3:**
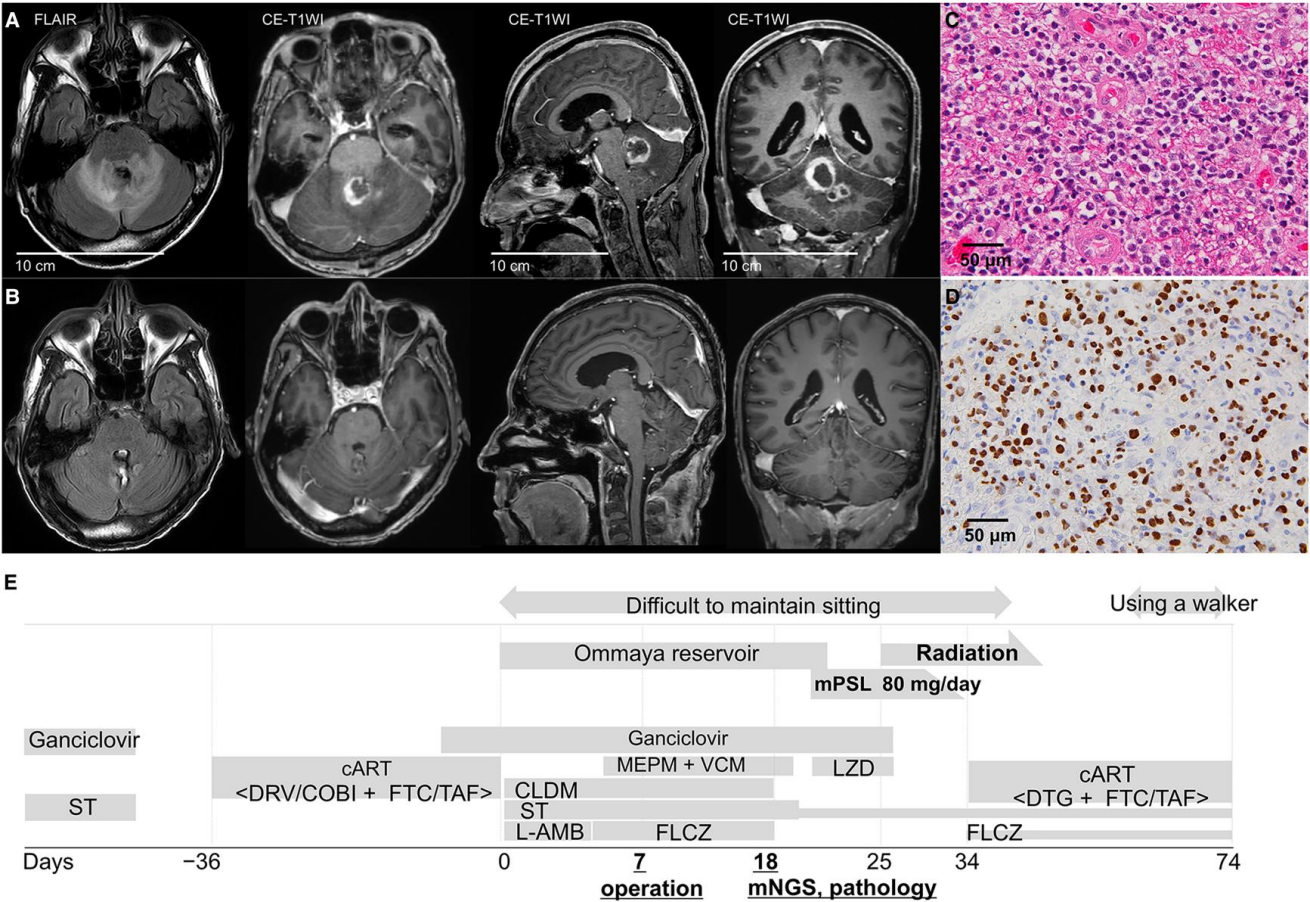
**Radiological, pathological and clinical course of PT-9 with EBV-positive diffuse large B-cell malignant lymphoma.** (**A**) FLAIR-MRI on Day 1 showing lesions in and around the cerebellar vermis; Ce-T1WI revealing nodular enhancing lesions in the same area. (**B**) Follow-up brain MRI on Day 70 showing complete resolution of the previously observed lesion. (**C**) HE staining showing the proliferation of atypical lymphocytic cells within the brain parenchyma. (**D**) EBER-ISH showing positive staining in the nuclei of EBV-infected cells. (**E**) The clinical course and treatments. EBER-ISH, EBV-encoded RNA-*in situ* hybridization; ST, sulphamethoxazole–trimethoprim; DRV/COBI, darunavir/cobicistat; FTC/TAF, emtricitabine/tenofovir alafenamide fumarate; DTG, dolutegravir; MEPM, meropenem; VCM, vancomycin hydrochloride; LZD, linezolid; CLDM, clindamycin; L-AMB, liposomal amphotericin B; FLCZ, fluconazole; mPSL, methylprednisolone; FLAIR, fluid attenuated inversion recovery; Ce-T1WIs, contrast-enhanced T1-weighted images.

A 50-year-old man was diagnosed with AIDS after experiencing fever and cytomegalovirus (CMV) sepsis following treatment for *Pneumocystis pneumonia*. After symptom resolution, he began combination antiretroviral therapy (cART), but severe side-effects required hospitalization. Brain MRI revealed a tumorous lesion in the cerebellar vermis and with subsequent acute obstructive hydrocephalus (Fig. 3A). Brain biopsy identified EBV-specific sequences, and pathological analysis revealed atypical lymphocytes (Fig. 3C and D). The patient was diagnosed with EBV-positive diffuse large B-cell lymphoma (DLBCL). Treatment with methylprednisolone, radiotherapy and cART resulted in significant improvement, restoring his ability to walk. By Day 70, a follow-up MRI showed complete resolution of the lesion (Fig. 3B).

### Toxoplasmic encephalitis (PT-13)

**Figure 4:**
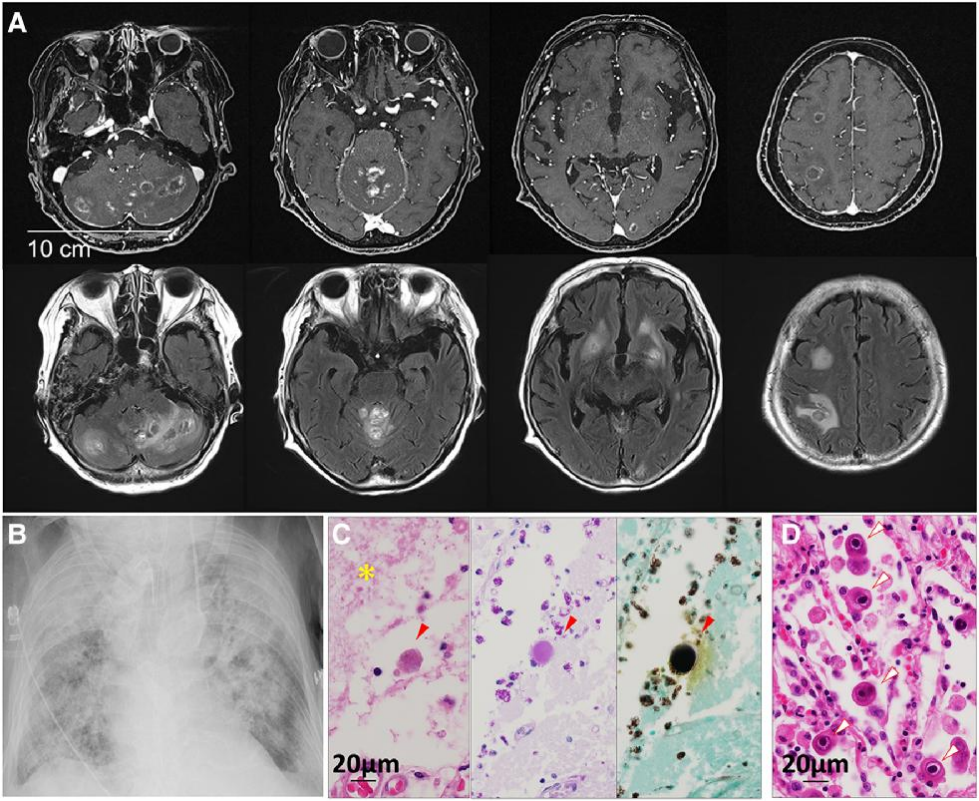
**Radiological and pathological findings in PT-13 with toxoplasma encephalitis and CMV pneumonia.** (**A**) Brain MRI (Day 22) shows lesions with ring enhancement in the cerebellar hemispheres, cerebellar mediastinum, left thalamus, bilateral basal ganglia, left occipital lobe and right deep white matter, as shown in the contrast-enhanced T1-weighted image (upper panel). These lesions show high signal intensity on FLAIR images (lower panel). (**B**) Chest X-ray (Day 79) reveals diffuse opacities in both lung fields, suggesting ARDS. (**C**) Histopathologic examination of the autopsy brain sample reveals sub-cortical necrosis (designated by asterisk) with neutrophilic infiltration and toxoplasma bradyzoites within cysts (designated with arrow heads) stained with HE, PAS and Grocott staining (from left to right). (**D**) Histopathologic examination of the autopsy lung sample shows numerous CMV-infected cells with characteristic nuclear inclusion bodies (designated with arrow heads). Bar = 20 μm.

An 82-year-old woman presented with loss of appetite and nausea. Gastrointestinal tests were normal, but brain MRI showed multiple nodular lesions (Fig. 4A). She had a history of rheumatoid arthritis and tested positive for anti-toxoplasma IgG, though CSF PCR was negative. Her condition worsened due to aspiration pneumonia (Fig. 4B), and she died on Day 52 from acute respiratory distress syndrome (ARDS) and pancytopenia. Post-mortem analysis utilizing mNGS detected eight reads associated with *T. gondii*, as well as two reads of CMV in brain tissue. Nested-PCR for *T. gondii was* positive (Supplementary Figs 3 and 4), and pathological analysis validated the presence of toxoplasma infection in the brain sample (Fig. 4C) and CMV infection in the lung (Fig. 4D).

### Recurrent herpes simplex encephalitis complicated by anti-N-methyl-D-aspartate receptor encephalitis (PT-17)

**Figure 5:**
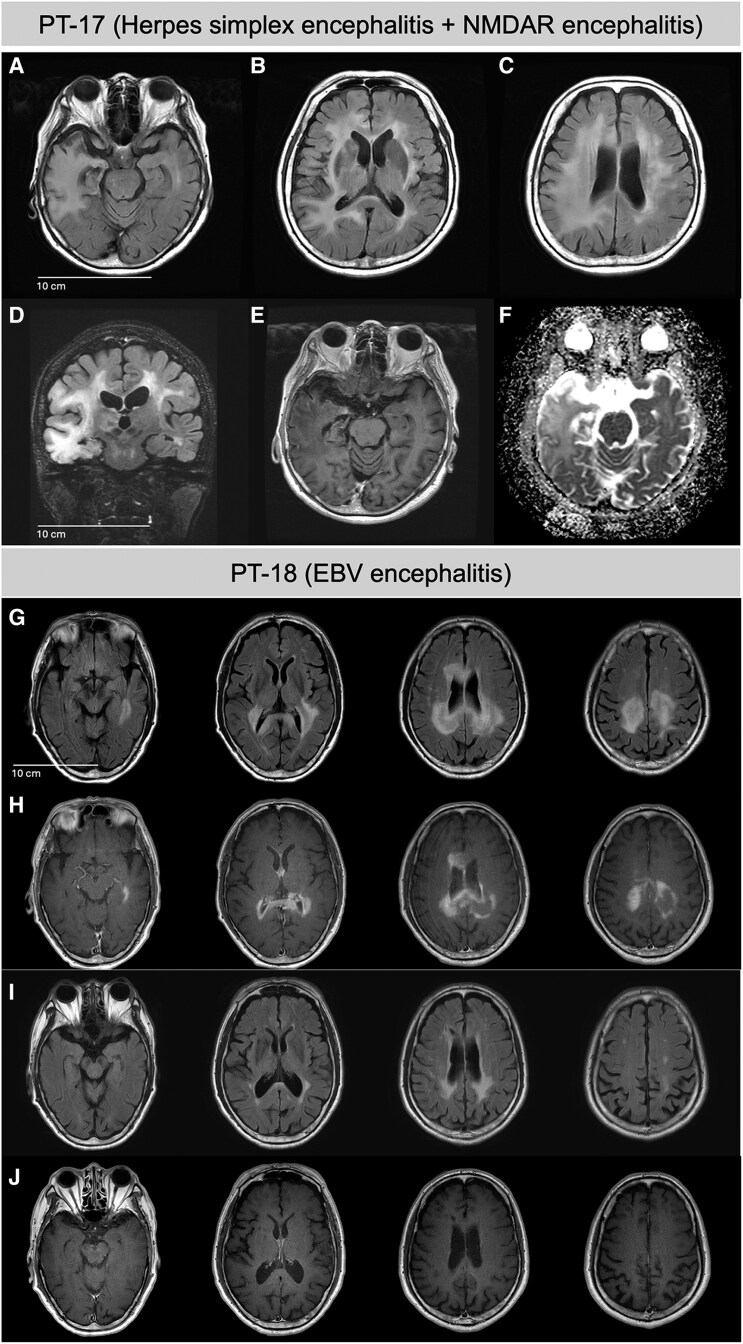
**Radiological findings of PT-17 (HSE and NMDAR encephalitis) and PT-18 (EBV encephalitis).** (**A–D**) In PT-17, horizontal and coronal sections of FLAIR-MRI (Day 60) showing right-sided dominant high signal in the temporal lobe, insula and periventricular white matter. (**E**) Contrast-enhanced T1-weighted image exhibits (Ce-T1WI; Day 60) slight enhancement effect on the meninges of the right temporal lobe, with no enhancement within the brain parenchyma. (**F**) Apparent diffusion coefficient map image (Day 300) revealing high signal in the right temporal lobe. (**G** and **H**) At Day 15 of PT-18, FLAIR-MRI illustrates high signal intensity in the periventricular region of the left lateral ventricle, cerebral peduncles and periventricular white matter of the right dominant side. Ce-T1WI displays enhancement corresponding to the abnormal signal area on FLAIR. (**I** and **J**) At Day 240, the FLAIR-MRI image depicts a smaller high-signal intensity area compared with previous scans, while Ce-T1WI shows no signal enhancement in previously identified regions. PT-17: (**A–F**); PT-18: (**G–J**). FLAIR, fluid attenuated inversion recovery; Ce-T1WIs: contrast-enhanced T1-weighted images.

A 70-year-old woman initially presented with dysarthria and dizziness at age 69 and was diagnosed with herpes simplex encephalitis (HSE) based on MRI abnormalities (Fig. 5A–E) and positive CSF IgM, despite negative HSV-DNA. Her symptoms temporarily improved with acyclovir, but worsened 2 months later, with MRI showing progression of white matter lesions. Brain biopsy revealed non-specific inflammation, ruling out malignancy. mNGS-brain analysis detected HSV-specific reads, prompting the resumption of acyclovir treatment. Positive anti-N-methyl-D-aspartate receptor (NMDAR) antibodies in the CSF indicated NMDAR encephalitis, and immunotherapy was added. Ten months later, she developed seizures, likely due to increased cortical perfusion in the right temporal lobe, as observed on arterial spin labeling (ASL) imaging (Fig. 5F). Anti-epileptic medications were initiated, resulting in partial symptom improvement.

### Epstein–Barr virus encephalitis (PT-18)

A 66-year-old male was referred to the hospital with a 10-day history of loss of appetite and memory impairment. Neurological examination showed no abnormalities except for a significant decrease in the Mini-Mental State Examination score (20/30), indicating cognitive decline with frontal lobe dysfunction. CSF analysis showed a protein level of 156 mg/dl, a cell count of 188 cells per µl and a glucose level of 58 mg/dl. Brain MRI revealed abnormal signals with contrast enhancement in the corpus callosum and periventricular white matter (Fig. 5G and H). The patient was treated with intravenous acyclovir, but its therapeutic effect was limited. Brain biopsy excluded the possibility of malignant lymphoma. Subsequent mNGS-brain analysis identified 11 reads corresponding to the EBV. Consequently, ganciclovir was included in the treatment regimen, resulting in symptom improvement, normalization of CSF findings and a reduction in the abnormal signals observed in brain MRI (Fig. 5I and J).

### Non-infectious CNS diseases

No pathogen-specific reads were detected in any of the 26 cases analysed using mNGS of brain samples. Subsequent pathological, CSF and serological analyses helped to diagnose 14 cases with non-infectious CNS diseases, including 7 neoplastic, 6 autoimmune or inflammatory disorders and 1 case of amyloid angiopathy (Supplementary Table 2). Pathological findings revealed DLBCL (PT-11 and PT-19) with CD20, Bcl-6 and MUM1 positivity; germinoma (PT-31) with PLAP and c-kit positivity; glioblastoma (PT-21) with morphological changes and Olig2 positivity; B-cell lymphoma (PT-28) with PAX5 positivity; leukaemia (PT-29) with blastoid cells positive for myeloperoxidase; primary CNS lymphoma (PT-20) without malignancy but positive for CD20 and CD5 and cerebral amyloid angiopathy (PT-33) confirmed by scarlet and Congo red staining.

CSF analysis supported the diagnoses of MOG antibody-associated encephalitis (PT-7 and PT-23) and GABAAR antibody-associated encephalitis (PT-25). Serological findings contributed to identifying rheumatoid meningitis with positive anti-CCP antibody (PT-8) and sarcoidosis with elevated serum angiotensin converting enzyme (ACE) levels (PT-3). Additionally, a patient suspected of acute disseminated encephalomyelitis (PT-32) showed demyelination on brain biopsy but was negative for MOG, AQP4 and GFAP antibodies.

### Unidentified cases

Among the other 12 cases where mNGS analysis of brain samples did not detect pathogen-specific reads, the underlying aetiology remained undetermined. However, it is noteworthy that none of these cases exhibited symptom progression after treatment with methylprednisolone pulse therapy (Supplementary Table 3).

## Discussion

Among the 33 brain samples analysed, mNGS successfully identified pathogen-specific sequences in seven cases (21.2%), aligning with diagnostic rates reported in previous studies, such as the 22% yield in a large-scale French observational study.^11^ Notably, in four cases (PT-1, PT-2, PT-6 and PT-18), pathogens undetected by initial conventional diagnostic tests were subsequently identified through mNGS-brain. This finding is consistent with our literature review, which revealed that mNGS-brain detected pathogens overlooked by traditional methods in 80% of reported cases. Moreover, mNGS was particularly effective in identifying pathogens such as archaea and amoebae, which are inherently challenging to detect using PCR-based assays. These results highlight the value of mNGS in expanding the diagnostic scope for encephalitis. Integrating mNGS findings with clinical and pathological evidence into routine workflows could significantly enhance aetiological assessment and guide targeted therapeutic interventions.

mNGS has proven to be an invaluable tool in complex clinical scenarios, particularly in cases involving co-infections and atypical presentations. For instance, in a patient with AIDS (PT-9), mNGS detected EBV, leading to the diagnosis of EBV-positive DLBCL. This diagnosis enabled the initiation of appropriate treatment with chemotherapy and radiotherapy. In another patient (PT-18), where malignant lymphoma was clinically suspected, mNGS revealed the presence of HSV-DNA, along with the detection of anti-NMDAR antibodies in the CSF, suggesting a co-diagnosis of recurrent HSV encephalitis and autoimmune encephalitis. These findings are consistent with prior studies reporting a 49% detection rate of anti-NMDAR antibodies in new cases of HSV encephalitis.^39^ Based on these findings, a targeted treatment combining antiviral and immunosuppressive therapies was administered, leading to partial improvement in the patient’s prognosis.

To ensure the reliability and accuracy of our mNGS findings, we implemented a comprehensive set of quality control measures. Stringent sterile techniques were employed throughout sample collection and handling to prevent contamination, alongside robust quality filtering, which retained only reads with a Phred score >30, ensuring high-quality data. To minimize cross-sample contamination, unique barcodes were incorporated, and rigorous cleaning protocols were strictly adhered to between sequencing runs, effectively reducing the risk of environmental contamination. The exclusion criteria employed further allowed us to eliminate the potential false-positive results. To validate the mNGS results, we conducted confirmatory tests using antibody assays and PCR, ensuring the accuracy and relevance of the detected pathogens. Rigorous quality control, careful interpretation and validation with complementary methods are essential to minimize false positives and false negatives.

The role of brain biopsy in unexplained encephalitis remains debated due to its invasive nature and variable diagnostic yield, despite its established utility in diagnosing suspected neoplastic lesions. A 2010 study found that biopsies led to treatment changes in only 8% of cases and significantly disease progression in just 4%.^3^ Furthermore, brain biopsies often fail to yield definitive diagnoses, with many cases classified as ENOS, posing challenges for pathologists and neurologists.^4^ However, a recent study reported longer survival in intensive care unit patients whose management was guided by biopsy findings.^40^ Combining mNGS-brain with brain pathology has shown promise in identifying rare pathogens missed by traditional methods.^41^ A systematic review reported low complication rates, with mortality ranging from 0% to 4% and symptomatic intracerebral haemorrhage rates between 0% and 8.6%.^42^ In this study, no serious complications were observed.

This study has several limitations. First, the small sample size (33 cases) and the limited scope of the literature review in Table 2 underscore the need for cautious interpretation of these findings. Larger studies are needed to better evaluate the clinical utility of the mNGS-brain approach. Second, preliminary verification was conducted with viral strains lacking cell walls or capsules, which are barriers to nucleic acid extraction in bacteria and fungi. Moreover, the vaccine strains did not contain human genomic material, unlike clinical samples, meaning these experiments may not fully reflect clinical conditions. Third, while the exclusion criteria effectively minimized false positives and false negatives in frozen brain biopsy samples, their reliability was reduced for CSF and paraffin-embedded samples (data not shown), emphasizing challenges in standardizing diagnostic criteria across different sample types.

Our inclusion criteria, designed to minimize the risk of overlooking encephalitis cases, inadvertently led to the inclusion of patients with non-infectious conditions, such as encephalopathy and malignancies. This expanded scope may have reduced the specificity of the analysis. We prioritized abnormal brain MRI findings, recognizing that encephalitis often presents with negative CSF cell counts, fever suppression due to prior steroid treatment and unrecognized headache in patients with impaired consciousness. Consequently, our cohort included a higher proportion of encephalitis cases, where fever and headache are less commonly observed compared with meningitis. More rigorous pre-test clinical assessments to evaluate the likelihood of infection could improve diagnostic accuracy.^11^ Despite the high cost and reliance on advanced sequencing platforms, which limit the accessibility of mNGS in many clinical settings, the technology demonstrated significant utility by excluding infectious aetiologies in over 40% of cases. This diagnostic precision facilitated targeted treatment for non-infectious conditions, with the potential to reduce unnecessary antibiotic use and overall healthcare costs.

## Conclusion

mNGS-brain represents a further advance in the diagnosis of encephalitis and related CNS diseases. To advance the integration of mNGS into clinical practice, the development of cost-effective platforms, such as pathogen-targeted NGS panels, is essential.^43^ These panels can provide comprehensive pathogen screening at a lower cost while preserving high diagnostic accuracy. Standardized workflows for sample collection, sequencing and data analysis are equally critical to ensure reproducibility and reliability across diverse clinical settings. Moreover, large-scale comparative studies evaluating mNGS against traditional diagnostic methods for specific pathogens could yield more robust statistical insights and inform the design of more efficient and targeted screening strategies in clinical practice.^44^ Combining mNGS with traditional pathology could uncover hidden pathogens, improving diagnosis and treatment, especially in complex cases.

## Supplemental material

Supplemental material is available at *Brain Communications* online.

## Supplementary Material

fcaf165_Supplementary_Data

## Data Availability

The raw mNGS-brain data from seven cases (PT-1, 2, 6, 9, 13, 17 and 18) have been deposited in the NCBI Sequence Read Archive (SRA) database (BioProject ID: PRJNA1034233; accession number: SAMN38055073–38055080). The other relevant data are available in the manuscript and the Supplementary material. Data not presented in the article are available upon reasonable request from the corresponding author.
