# Supplementary material for: Brain biopsy and metagenomic sequencing enhance aetiological diagnosis of encephalitis
Source: Brain Commun. 2025 Apr 28;7(3):fcaf165. doi: 10.1093/braincomms/fcaf165 (PMC12059644; doi:10.1093/braincomms/fcaf165)
Supplement: fcaf165_Supplementary_Data [file fcaf165_supplementary_data.docx]

**Supplementary material**

**List of Contents**

**PubMed search strategy for mNGS case reports**…..…………...…..………………………..…….....… 2

**Supplementary Fig. 1: Eight facilities involved in this study**…..…………………………………....... 3

**Supplementary Fig. 2: Preliminary analysis of vaccine strains through mNGS**……………………... 4

**Supplementary Fig. 3:** **Detection of the Toxoplasma gondii 18S-rDNA by nested-PCR (PT-13)**….... 5

**Supplementary Fig. 4: Uncropped blot corresponding to Supplementary Fig. 3**……..…...….. 5

**Supplementary Table 1: Literature review of shotgun mNGS for brain samples from patients with encephalitis**………………………………………………………………………………………………... 6

**Supplementary Table 2: Summary of 14 cases with non-infectious CNS diseases**………………….. 8

**Supplementary Table 3: Summary of 12 cases in the unidentified group**…………………………… 13

**References**………………………………………………………………………………………………… 14

**PubMed search strategy for literature review using mNGS-brain**

(((("brain"[MeSH Terms] OR "brain"[tiab]) AND (“infections”[Mesh Term] OR ”infect*"[tiab] )) OR (("encephalitis"[MeSH Terms]) OR "encephalities"[tiab] ))) AND ((“Metagenomics”[Mesh Term] OR ”High-Throughput Nucleotide Sequencing”[Mesh Term] OR “pyrosequencing"[tiab] OR "deep sequencing"[tiab] OR "next generation sequencing"[tiab] OR "metagenom*"[tiab] OR "metatranscriptom*"[tiab]))) AND (("biopsy"[Mesh Terms] OR "biops*"[tiab] OR "brain tissue*"[tiab] OR "brain sample*"[tiab] OR ("autopsy"[MeSH Terms] OR "autops*"[tiab]) OR "postmortem*"[tiab] OR “fatal outcome” [Mesh Term]))

**
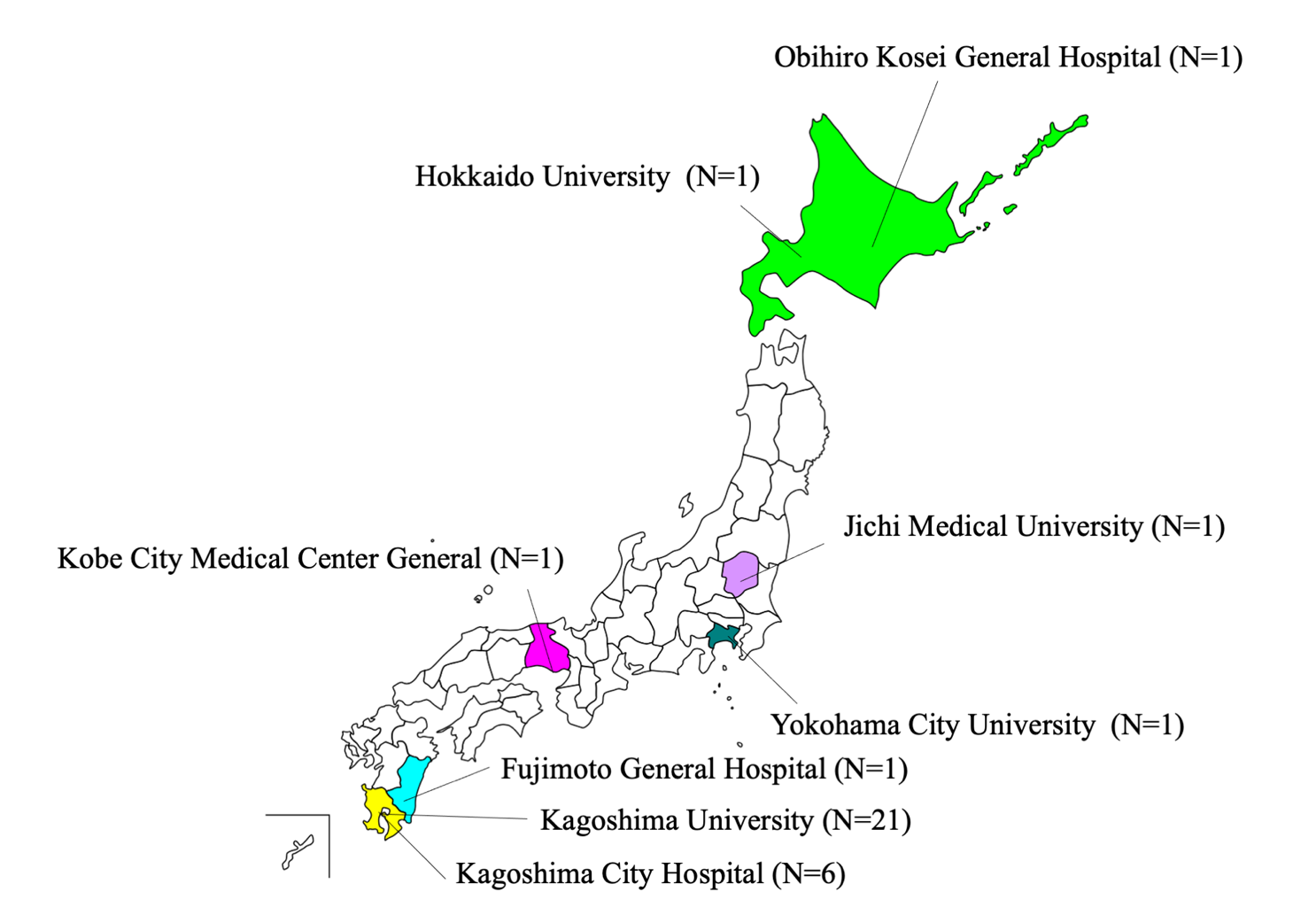
**

**Supplementary Fig. 1: Eight facilities involved in this study**

This map displaying the eight Japanese medical facilities involved in this study, along with the corresponding number of cases.

**
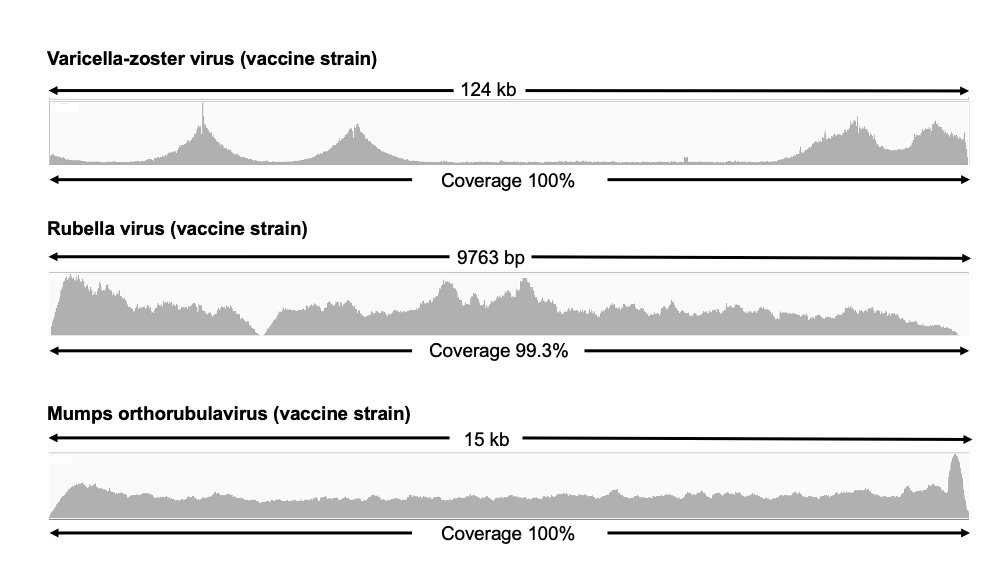
**

**Supplementary Fig. 2: Preliminary analysis of vaccine strains through mNGS**

Preliminary mNGS analyses indicating sufficient sequencing coverage on vaccine strains of varicella-zoster, rubella, and mumps orthorubulavirus.

**
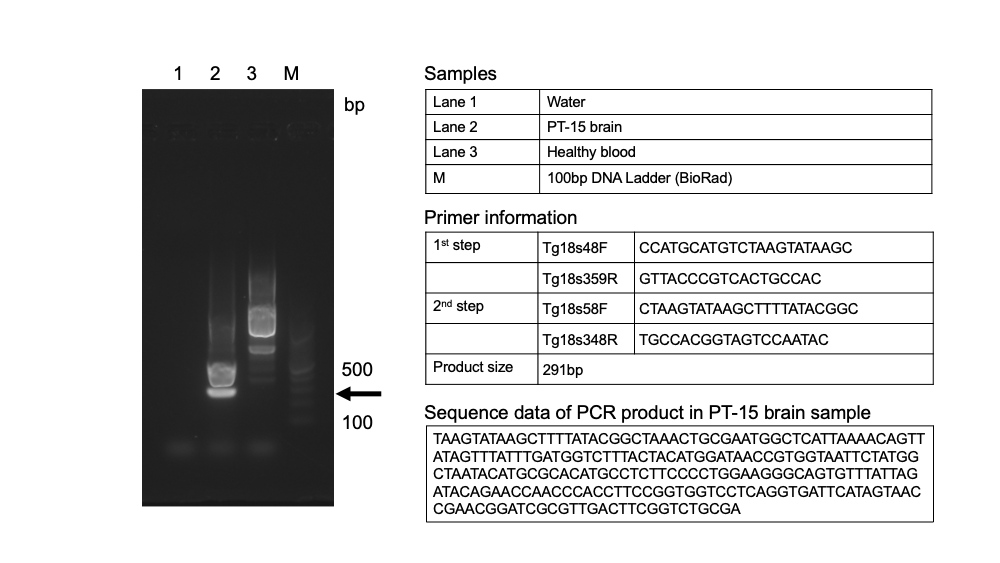
**

**Supplementary Fig. 3: Detection of the *Toxoplasma gondii* 18S ribosomal DNA by nested-PCR (PT-13)**

DNA isolated from frozen brain tissue was subjected to a specific nested PCR targeting the *Toxoplasma gondii* nucleotide sequences.^1^ Subsequently, the resulting PCR products (290 base pairs) were processed to Sanger sequencing employing the ABI PRISM 3500xl Genetic Analyzer (Applied Biosystems). The arrow indicates a positive PCR result and has been subsequently validated by sanger sequencing. The corresponding full-size and uncropped image is provided in the supplementary material Figure 4-Uncropped.


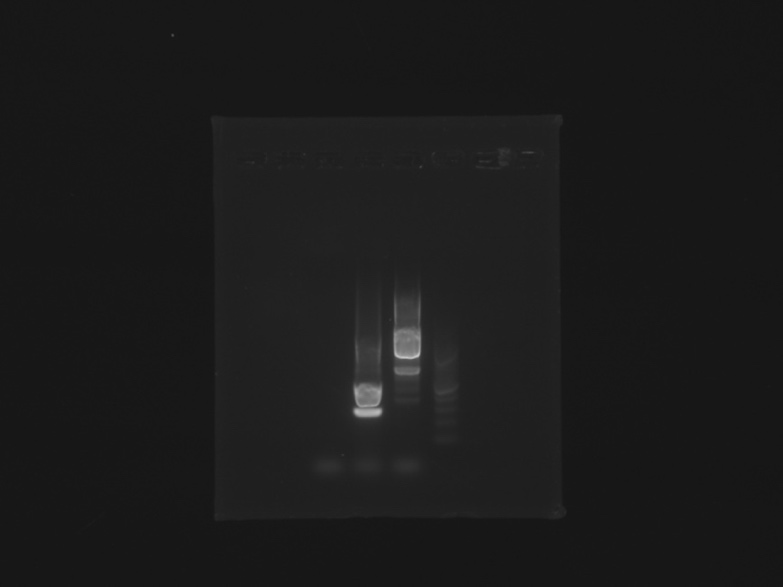


**Supplementary Fig. 4: Uncropped blot corresponding to Supplementary Figure 3**

**Supplementary Table 1: Literature review of shotgun mNGS for brain samples from patients with encephalitis**

| Citation | Pathogen | Total reads | Reads without human genome | Reads of pathogen | Initial test | Confirmatory test | Diagnosis | Treatment | Outcome |
| --- | --- | --- | --- | --- | --- | --- | --- | --- | --- |
| Quan et al. (2010)^2^ | Astrovirus | 102,000 | Not reported | 12 | Not detected | PCR and IH | Astrovirus encephalitis | None | Postmortem sample |
| Chan et al. (2014)^3^ | Measles virus | 76,983,381 | 877,782 | 1067 | Detected (Pathology) | PCR | SSPE | None | Brain Banks sample |
|  | Measles virus | 75,031,555 | 199,666 | 46434 | Detected (Pathology) | PCR | SSPE | None | Brain Banks sample |
|  | HSV1 | 60,275,421 | 786,777 | 10 | Detected (Pathology) | PCR | HSV1 encephalitis | None | Brain Banks sample |
|  | HSV1 | 92,699,696 | 758,199 | 9 | Not detected | PCR | HSV1 encephalitis | None | Brain Banks sample |
|  | HSV1 | 111,113,603 | 833,085 | 35 | Detected (Pathology) | PCR | HSV1 encephalitis | None | Brain Banks sample |
| Brown et al. (2015)^4^ | Astrovirus | 18,877,676 | 77,915 | 46 | Not detected | PCR, IH and EM | Astrovirus encephalitis | None | Died |
| ﻿Frémond et al.(2015)^5^ | Astrovirus | 75,600,000 | Not reported | 15 contigs | Not detected | PCR | Astrovirus encephalitis | IVIg, mPSL, ribavirin, PEG-IFN alpha-2b | Partialy improvement |
| Greninger et al.(2015)^6^ | *Balamuthia mandrillaris* | 3,482,508 | 347,926 | 30 | Not detected | PCR and Pathology | Primary amoebic meningoencephalitis | Miltefosine did not arrive in time | Died |
| Naccache et al.(2015)^7^ | Astrovirus | 381,014,716 | Not reported | 1612 | Not detected | PCR and IH | Astrovirus encephalitis | Ribavirin and immunoglobulin | Died |
| Lum et al.(2016)^8^ | Astrovirus | Not reported | Not reported | Not reported | Not detected | PCR | Astrovirus encephalitis | Immune reconstitution | Postmortem sample |
| Morfopoulou et al. (2016)^9^ | HCoV-OC43 | 64,000,000 | 1,400,000 | 1,000,000 | Not detected | PCR and IH | HCoV-OC43 encephalitis | ﻿Unconditioned cord-blood transplantation | Died |
| Salzberg et al. (2016)^10^ | JC polyomavirus | 26,919,065 | Not reported | 8,944 | Detected (PCR) | Pathology and IH | PML | Not reported | Not reported |
|  | *M. tuberculosis* | 13,990,253 | Not reported | 15 | Not detected | Pathology | Granuloma, Tuberculosis | Responded rapidly to antituberculous treatment | Improvement |
|  | EBV | 21,319,274 | Not reported | 18 | Not detected | ISH | EBV encephalitis | Not reported | Not reported |
| Morfopoulou et al. (2017)^11^ | Mumps vaccine strain | 110,000,000 | 1,000,000 | 77,624 | Not detected | PCR and IH | Chronic encephalitis caused by mumps vaccine stain | ﻿Broad-spectrum antibiotics, aciclovir, ganciclovir, antifungal therapy, IVIg and mPSL | Died |
| Wilson et al. (2017)^12^ | Cache Valley virus | 13,661,871 | Not reported | 2 | Not detected | PCR and IH | Chronic viral meningoencephalitis | Antibiotics, aciclovir and IVIg | Died |
| Lipowski et al. (2017)^13^ | Tick-borne encephalitis virus | 39,064,947 | 193,764 | 9 | Not detected | PCR | Tick-borne encephalitis | Antibiotics and aciclovir | Postmortem sample |
|  | Tick-borne encephalitis virus | 33,133,366 | 54,376 | 988 | Not detected | PCR | Tick-borne encephalitis | None | Postmortem sample |
| Osterman et al. (2020)^14^ | HSV1 | 2,186,683 | Not reported | 490 | Not detected | PCR | HSV1 encephalitis | Antibiotics, aciclovir and mPSL | Postmortem sample |
| Normandin et al. (2020)^15^ | Powassan virus | Not reported | Not reported | Not reported | Not detected | PCR and IH | Fatal encephalitis | Not reported | Postmortem sample |
| Rodriguez et al. (2020)^16^ | Measles virus | Not reported | Not reported | >4,800,000 | Not detected | PCR | Measles inclusion-body encephalitis | Antiepileptic and ﻿supportive treatment | Died |
| Tuddenham R, et al. (2020)^17^ | Human pegivirus | 8,557,321 |  | 4 | Not detected | PCR | Enchephalitis (Etiology unknown) | Antibiotics, mPSL, PE, cyclophosphamide | Improvement |
| Nilsson et al. (2020)^18^ | HCoV-OC43 | 24,000,000 | Not reported | 300,000 | Not detected | PCR | HCoV-OC43 encephalitis | Lopinavir boosted by ritonavir | Died |
| Pérot P, et al. (2021)^19^ | Umbre Orthobunyavirus | 116,800,000 | Not reported | 563 | Not detected | PCR and ISH | Lethal Encephalitis | Antidepressants, benzodiazepines, or neuroleptics | Postmortem sample |
|  | Umbre Orthobunyavirus | 40,100,000 | Not reported | 2,029 | Not detected | PCR and ISH | Lethal Encephalitis | Antibiotics, aciclovir, IVIg, mPSL, and PE | Postmortem sample |
| Howard-Jones et al. (2022)^20^ | Japanese encephalitis virus | Not reported | Not reported | Not reported | Not detected | PCR and IgM | Japanese encephalitis | Not reported | Not reported |
| Regnault B, et al. (2022)^21^ | European Bat Lyssavirus Type 1 | 94,000,000 | Not reported | (90% coverage of reference genome) | Not detected | PCR and EM | Lethal Encephalitis | Antibiotics, aciclovir, IVIg, mPSL | Died |
| Maamary J, et al. (2023)^22^ | ﻿Japanese encephalitis virus | Not reported | Not reported | Not reported | Not detected | IgM | Japanese encephalitis | Antibiotics, aciclovir | Postmortem sample |
| Gould et al. (2023)^23^ | Yellow fever vaccine virus | Not reported | Not reported | 15 | Detected (PCR, IgM) | ISH | Encephalitis caused by yellow fever vaccine stain | IVIg, mPSL and PE | Postmortem sample |
| Piantadosi et al. (2023)^24^ | JC polyomavirus | 14,789,558 | Not reported | 19 | Not detected | PCR | PML suspected | Not reported | Disease progression |
| Guo et al. (2024)^25^ | *Schistosoma japonicum* | Not reported | Not reported | Not reported | Not detected | Pathology | Cerebral schistosomiasis | praziquantel | Improvement |

HSV-1: herpes simplex virus type 1, HCoV: human coronavirus, EBV: Epstein-Barr virus, PCR: polymerase chain reaction, SSPE: subacute sclerosing panencephalitis, PML: progressive multifocal leukoencephalopathy, IH: immunohistochemistry, ISH: in situ hybridization, mPSL: methylprednisolone, IVIg: intravenous immunoglobulin, PE: plasma exchange

**Supplementary Table 2: Summary of 14 cases with non-infectious CNS diseases**

| Case | Age | Main symptoms | MRI findings | CSF- Protein (mg/dl) | CSF-Cell (/µl) | CSF-Glucose (mg/µl) | Duration  Onset–mNGS | Brain frozen sample | mNGS-Total reads | mNGS-Suspected pathogen | Main test results | Diagnosis | Treatment | Clinical course |
| --- | --- | --- | --- | --- | --- | --- | --- | --- | --- | --- | --- | --- | --- | --- |
| PT-3 | 70s | Left hemiplegia | Right white matter lesions, midline shift and hemosiderin deposits. | 118 | 13 | 86 | 44 days | Biopsied | 12,340,936 | None | Brain pathology: granulomatous lesions with giant Langerhans cells    Elevated serum-ACE | Sarcoidosis | Prednisolone and minocycline | Improvement |
| PT-7 | 30s | Abnormal sensation in extremities, dysuria and dysphagia | T2-WI high signal in bilateral subcortical white matter, thalamus, cerebral legs, medulla oblongata and cerebellar hemispheres. Extensive lesion with open ring contrast effect in the right frontal lobe. | 52.2 | 32 | 57 | 3 years | Biopsied | 8,886.351 | None | Brain pathology: demyelination  CSF-MOG antibody (+) | MOG antibody-associated encephalitis | Prednisolone, selective PE, azathioprine, tacrolimus and levetiracetam | Improvement |
| PT-8 | 80s | Fever up  and disturbance of consciousness | FLAIR high signal and contrast effect on the arachnoid and dura mater of the left parietal lobe. Proximal cortical thickening and T2-WI high signal in the subcortical white matter. | 40.5 | 5 | 44 | 9 months | Biopsied | 18,799,350 | None | Serum-CCP antibody (+) | Rheumatoid meningitis | Prednisolone | Partial improvement |
| PT-11 | 40s | Headache and convulsive seizures | T2-WI high signal in left frontal lobe with contrast effect. | 120 | 3 | 86 | 56 days | Biopsied | 2,902,616 | None | Brain pathology: diffuse proliferation of atypical cells  Immunostaining: CD20 (+), CD79a (+), CD3 (-), CD4 (-), CD8 (-), CD10(-), Bcl-6 (+), MUM1 (+), and EBER-ISH (-) | DLBCL | Radiation | Died |
| PT-19 | 50s | Disorientation and apathy | T2-WI high signal in bilateral frontal lobes, temporal lobes, caudate nucleus, and dorsal brainstem. | 98 | 76 | 67 | 3 months | Biopsied | 3,789,474 | None | Brain pathology: diffuse proliferation of atypical cells  Immunostaining: CD20 (+), CD10 (+), Bcl-6 (+), and MUM1 (+) | DLBCL | R-MTX and R-MPV | Improvement |
| PT-20 | 60s | Disturbance of consciousness | FLAIR high signal in the brain surface and cortex of the bilateral cerebral hemispheres, brainstem, and periventricular ependyma. Enhancement of the oculomotor, trigeminal, facial, and auditory nerves. | 512 | 111 | 50 | 58 days | Biopsied | 2,555,016 | None | Brain pathology: no malignancy  CSF cytology: atypical cell  CSF flowcytometry: CD20 (+) and CD5 (+) | PCNSL suspected | MTX, Ara-C and prednisolone | Disease progression |
| PT-21 | 60s | Dementia | The right caudate nucleus, right frontal lobe white matter, and periventricular white matter around the trigone of the right lateral ventricle exhibit high signal intensity on T2-WI, with partial extension into the cortex. Mass effect is also observed. No evident contrast enhancement effect is identified. | 64.8 | 1 | 68 | 10 years | Biopsied | 2,683,406 | None | Brain pathology: hematoxylin and eosin staining reveal nuclear atypia, variable cell density, and scattered mitotic figures in glial cells.  Atypical cells with enlarged nuclei exhibit Olig2 positivity.  MIB-1 positive ratio: 15% | Diffuse astrocytic glioma with molecular feature of glioblastoma | TMZ, IMRT and Bev | Disease progression |
| PT-23 | 40s | Dementia | T2-WI high signal nodular lesions and edematous changes are observed around the right lateral ventricular inferior horn and the left lateral ventricular trigone. The lesions demonstrate contrast enhancement with an open-ring pattern. | N.R. | N.R. | N.R. | 2 months | Biopsied | 2,698,196 | None | Brain pathology: demyelination  CSF-MOG antibody (+) | MOG antibody-associated encephalitis | Prednisolone, IAPP, azathioprine and mycophenolate mofetil | Partial improvement  Recurrence |
| PT-25 | 50s | Abnormal behavior | T2-WI high signal is observed in the cortex and subcortical regions extending from the bilateral frontal lobes, right temporal lobe, and left parietal to occipital lobes. The cortex are swollen. | 85.9 | 4 | 165 | 43 days | Biopsied | 3,547,546 | None | Serum-GABAAR antibody (+) | GABAAR antibody-associated encephalitis | Prednisolone, DFPP, azathioprine, IVCY and levetiracetam | Disease progression |
| PT-28 | 40s | Convulsive seizures | T2-WI reveals high signal intensity on the right temporal pole, right insular cortex, subcortical white matter, and deep white matter of the right frontal lobe. Contrast-enhanced imaging demonstrates enhancement effects in the meninges and pia mater of the right frontal and temporal lobes. | 223 | 125 | 156 | 10 months | Biopsied | 5,313,328 | None | Brain pathology: perivascular infiltration of CD5 (-), CD10 (-), CD20 (+), PAX5 (+), a low-grade B-cell lymphoma. | Intraparenchymal CNS MALT lymphoma | R-MTX | Disease progression |
| PT-29 | 80s | Abnormal sensation in right upper extremity | Enhancing tumor lesions are identified in the left postcentral gyrus and the right inferior frontal gyrus, exhibiting contrast enhancement. The periphery of the lesions demonstrates extensive high signal intensity on T2-WI. | 103 | 3 | 60 | 24 days | Biopsied | 3,858,040 | None | Brain pathology: most blastoid cells infiltrate diffusely and around blood vessels, showing positivity for myeloperoxidase. | Leukemia | Unknown | Unknown |
| PT-31 | 10s | Headache and vomiting | Two distinct occupying lesions demonstrating a ring-enhancing pattern in the left frontal lobe are observed. The lesion peripheries exhibit low signal intensity on T2-WI, with a wide surrounding area demonstrating high signal intensity on T2-WI. | 105.7 | 42 | 56 | 30 days | Biopsied | 5,895,310 | None | Brain pathology: diffuse proliferation of atypical cells  Immunostaining: PLAP (+) and c-kit (+)  MIB-1 positive ratio: 80% | Germinoma | Surgical resection, radiation and CARE | Improvement |
| PT-32 | 40s | Abnormal sensation in right cheek and right upper limb | Multiple T2-WI high signal intensities and distinct contrast enhancement effects are observed in the pons, bilateral temporal lobes, right frontal lobe, corpus callosum, and spinal cord. | 27 | 2 | 53 | 39 days | Biopsied | 4,737,146 | None | Brain pathology: demyelination  CSF-MOG/AQP4/GFAP antibodies (-) | ADEM | Prednisolone | Partial improvement  Recurrence |
| PT-33 | 80s | Disturbance of consciousness | Widespread T2-WI high signal intensity and numerous microhemorrhages are observed in the cortical and subcortical white matter of both lateral temporal and parietal lobes. | 66.5 | 1 | 72 | 31 days | Biopsied | 4,919,430 | None | Brain pathology: mild wall thickening primarily within the cortical small blood vessel walls with hemosiderin deposition and corpora amylacea.  Direct fast scarlet staining and Congo red staining are both positive. | Cerebral amyloid angiopathy | Prednisolone | Died |

PT: patient, T2-WI: T2-weighted image, FLAIR: fluid-attenuated inversion recovery, ACE: angiotensin-converting enzyme, MOG: myelin oligodendrocyte glycoprotein, CCP: cyclic citrullinated peptide, CD: cluster of differentiation, MUM1: Multiple myeloma oncogene 1, EBER-ISH: EBV-encoded small RNA in situ hybridization, MALT: mucosa-associated lymphoid tissue, PLAP: placental alkaline phosphatase, AQP4: aquaporin 4, GFAP: glial fibrillary acidic protein, DLBCL: diffuse large B-cell lymphoma, PCNSL: primary central nervous system lymphoma, R-MTX: high-dose MTX plus rituximab, R-MPV: high-dose MTX plus rituximab, vincristine, and procarbazine, TMZ: temodar, IMRT: intensity modulated radiation therapy, Bev: bevacizumab, IAPP: immunoadsorption plasmapheresis, DFPP: double filtration plasmapheresis, IVCY: intravenous cyclophosphamide, CARE: carboplatin and etoposide

**Supplementary Table 3: Summary of 12 cases in the unidentified group**

| Case | Age | Sample | CSF-Protein(mg/dl) | CSF-  Cell (/µl) | CSF-  Glucose (mg/dl) | Duration Onset-mNGS (day) | mNGS-Total reads | Suspected final diagnosis | Treatment | Clinical course |
| --- | --- | --- | --- | --- | --- | --- | --- | --- | --- | --- |
| PT-4 | 80s | Biopsied brain frozen sample | 97.8 | 2 | 62 | 1145 | 9,704,870 | Neuro-sarcoidosis | Prednisolone and minocycline | Improvement |
| PT-5 | 50s | Biopsied brain frozen sample | 74.1 | 15 | 45 | 532 | 15,150,646 | Chronic neuro-Behçet disease | Prednisolone, colchicine and MTX | No improvement |
| PT-10 | 60s | Biopsied brain frozen sample | 58.1 | 1 | 54 | 64 | 6,902,396 | Post-transplant lympho­proliferative disorder | Radiation | Died |
| PT-12 | 60s | Postmortem brain frozen sample | 55 | 1 | 158 | 83 | 6,244,574 | Mitochondrial disease  Leukoencephalopathy | Unknown | Unknown |
| PT-14 | 70s | Biopsied brain frozen sample | 123 | 1 | 71 | 162 | 2,870,648 | Leukoencephalopathy Malignant lymphoma, PML | Unknown | Died |
| PT-15 | 80s | Postmortem brain frozen sample | 119 | 52 | 78 | 574 | 5,149,380 | Multiple cerebral infarction + meningitis | Unknown | Died (d/t sepsis) |
| PT-16 | 60s | Biopsied brain frozen sample | 135.9 | 1 | 67 | 248 | 3,189,898 | Lymphoproliferative disorder | Prednisolone | Partial improvement |
| PT-22 | 50s | Biopsied brain frozen sample | 75.7 | 11 | 60 | 63 | 7,504,574 | Demyelinating disease | Unknown | Unknown |
| PT-24 | 20s | Biopsied brain frozen sample | 39 | 2 | 49 | 118 | 3,594,852 | Demyelinating disease | Prednisolone, levetiracetam, rituximab, IVCY and plasma exchange | No improvement |
| PT-26 | 50s | Biopsied brain frozen sample | 200 | 52 | 70 | 115 | 6,197,970 | Autoimmune encephalitis | Prednisolone, IVIg, rituximab and plasma exchange | Partial improvement |
| PT-27 | 30s | Biopsied brain FFPE sample | 34 | 38 | 84 | 58 | 7,000,662 | Autoimmune encephalitis | Acyclovir, antibiotics, prednisolone, IVIg, and plasma exchange | Partial improvement |
| PT-30 | 70s | Biopsied brain frozen sample | 468 | 580 | 133 | 127 | 3,858,040 | Malignant lymphoma | Antibiotics and prednisolone | Died |

FFPE: Formalin-Fixed Paraffin-Embedded, MTX: methotrexate, IVCY: intravenous cyclophosphamide, IVIg: intravenous immunoglobulin

**References**

1. da Silva RC, Su C, Langoni H. First identification of Sarcocystis tenella (Railliet, 1886) Moulé, 1886 (Protozoa: Apicomplexa) by PCR in naturally infected sheep from Brazil. *Vet Parasitol*. 2009;165(3-4):332-336. doi:10.1016/j.vetpar.2009.07.016

2. Quan PL, Wagner TA, Briese T, et al. Astrovirus encephalitis in boy with X-linked agammaglobulinemia. *Emerg Infect Dis*. 2010;16(6):918-925. doi:10.3201/eid1606.091536

3. Chan BK, Wilson T, Fischer KF, Kriesel JD. Deep sequencing to identify the causes of viral encephalitis. *PLoS One*. 2014;9(4). doi:10.1371/journal.pone.0093993

4. Brown JR, Morfopoulou S, Hubb J, et al. Astrovirus VA1/HMO-C: An increasingly recognized neurotropic pathogen in immunocompromised patients. *Clinical Infectious Diseases*. 2015;60(6):881-888. doi:10.1093/cid/ciu940

5. Frémond ML, Pérot P, Muth E, et al. Next-generation sequencing for diagnosis and tailored therapy: A case report of astrovirus- associated progressive encephalitis. *J Pediatric Infect Dis Soc*. 2015;4(3):e53-e57. doi:10.1093/jpids/piv040

6. Greninger AL, Messacar K, Dunnebacke T, et al. Clinical metagenomic identification of Balamuthia mandrillaris encephalitis and assembly of the draft genome: The continuing case for reference genome sequencing. *Genome Med*. 2015;7(1):1-14. doi:10.1186/s13073-015-0235-2

7. Naccache SN, Peggs KS, Mattes FM, et al. Diagnosis of neuroinvasive astrovirus infection in an immunocompromised adult with encephalitis by unbiased next-generation sequencing. *Clinical Infectious Diseases*. 2015;60(6):919-923. doi:10.1093/cid/ciu912

8. Lum SH, Turner A, Guiver M, et al. An emerging opportunistic infection: fatal astrovirus (VA1/HMO-C) encephalitis in a pediatric stem cell transplant recipient. *Transplant Infectious Disease*. 2016;18(6):960-964. doi:10.1111/tid.12607

9. Morfopoulou S, Brown JR, Davies EG, et al. Human Coronavirus OC43 Associated with Fatal Encephalitis. *New England Journal of Medicine*. 2016;375(5):497-498. doi:10.1056/nejmc1509458

10. Salzberg SL, Breitwieser FP, Kumar A, et al. Next-generation sequencing in neuropathologic diagnosis of infections of the nervous system. *Neurol Neuroimmunol Neuroinflamm*. 2016;3(4):1-9. doi:10.1212/NXI.0000000000000251

11. Morfopoulou S, Mee ET, Connaughton SM, et al. Deep sequencing reveals persistence of cell-associated mumps vaccine virus in chronic encephalitis. *Acta Neuropathol*. 2017;133(1):139-147. doi:10.1007/s00401-016-1629-y

12. Wilson MR, Suan D, Duggins A, et al. A novel cause of chronic viral meningoencephalitis: Cache Valley virus. *Ann Neurol*. 2017;82(1):105-114. doi:10.1002/ana.24982

13. Lipowski D, Popiel M, Perlejewski K, et al. A cluster of fatal Tick-borne encephalitis virus infection in organ transplant setting. *Journal of Infectious Diseases*. 2017;215(6):896-901. doi:10.1093/infdis/jix040

14. Osterman A, Ruf VC, Domingo C, et al. Travel-Associated neurological disease terminated in a postmortem diagnosed atypical HSV-1 encephalitis after high-dose steroid therapy-a case report. *BMC Infect Dis*. 2020;20(1):1-9. doi:10.1186/s12879-020-4859-5

15. Normandin E, Solomon IH, Zamirpour S, et al. Powassan virus neuropathology and genomic diversity in patients with fatal encephalitis. *Open Forum Infect Dis*. 2020;7(10):1-10. doi:10.1093/ofid/ofaa392

16. Rodriguez C, Gouilh M, Weiss N, et al. Fatal Measles Inclusion-Body encephalitis in adult with untreated AIDS, France. *Emerg Infect Dis*. 2020;26(9):2231-2234. doi:10.3201/eid2609.200366

17. Tuddenham R, Eden JS, Gilbey T, et al. Human pegivirus in brain tissue of a patient with encephalitis. *Diagn Microbiol Infect Dis*. 2020;96(2):114898. doi:10.1016/j.diagmicrobio.2019.114898

18. Nilsson A, Edner N, Albert J, Ternhag A. Fatal encephalitis associated with coronavirus OC43 in an immunocompromised child. *Infect Dis*. 2020;52(6):419-422. doi:10.1080/23744235.2020.1729403

19. Pérot P, Bielle F, Bigot T, et al. Identification of Umbre Orthobunyavirus as a Novel Zoonotic Virus Responsible for Lethal Encephalitis in 2 French Patients with Hypogammaglobulinemia. 2021;72(10):1701-1708. doi:10.1093/cid/ciaa308

20. Howard-Jones AR, Pham D, Jeoffreys N, et al. Emerging Genotype IV Japanese Encephalitis Virus Outbreak in New South Wales, Australia. *Viruses*. 2022;14(9):1-12. doi:10.3390/v14091853

21. Regnault B, Evrard B, Plu I, et al. First Case of Lethal Encephalitis in Western Europe Due to European Bat Lyssavirus Type 1. *Clinical Infectious Diseases*. 2022;74(3):461-466. doi:10.1093/cid/ciab443

22. Maamary J, Maddocks S, Barnett Y, et al. New Detection of Locally Acquired Japanese Encephalitis Virus Using Clinical Metagenomics, New South Wales, Australia. *Emerg Infect Dis*. 2023;29(3):627-630. doi:10.3201/eid2903.220632

23. Gould C V., Free RJ, Bhatnagar J, et al. Transmission of yellow fever vaccine virus through blood transfusion and organ transplantation in the USA in 2021: report of an investigation. *Lancet Microbe*. 2023;4(9):e711-e721. doi:10.1016/S2666-5247(23)00170-2

24. Piantadosi A, Shariatzadeh N, Bombin A, et al. Double-stranded RNA immunohistochemistry as a screening tool for viral encephalitis. *Am J Clin Pathol*. 2023;160(2):210-219. doi:10.1093/ajcp/aqad039

25. Guo Y, Zhang J, Chai R, et al. Cerebral schistosomiasis in a 3-year-old girl due to Schistosoma japonicum: a case report. *Front Immunol*. 2024;15(December):1-6. doi:10.3389/fimmu.2024.1502627
